# Towards a metagenomic understanding on enhanced biomethane production from waste activated sludge after pH 10 pretreatment

**DOI:** 10.1186/1754-6834-6-38

**Published:** 2013-03-19

**Authors:** Mabel Ting Wong, Dong Zhang, Jun Li, Raymond Kin Hi Hui, Hein Min Tun, Manreetpal Singh Brar, Tae-Jin Park, Yinguang Chen, Frederick C Leung

**Affiliations:** 1 5 N01, Kadoorie Biological Sciences Building, School of Biological Sciences, The University of Hong Kong, Pokfulam Road, Hong Kong, Hong Kong; 2 State Key Laboratory of Pollution Control and Resources Reuse, School of Environmental Science and Engineering, Tongji University, 1239 Siping Road, Shanghai, 200092, China; 3 Bioinformatics Center, Nanjing Agricultural University, Nanjing, China

**Keywords:** Wastewater treatment plant, Sludge, Pretreatment, Renewable energy, Biomethane, Pyrosequencing, Metagenomic

## Abstract

**Background:**

Understanding the effects of pretreatment on anaerobic digestion of sludge waste from wastewater treatment plants is becoming increasingly important, as impetus moves towards the utilization of sludge for renewable energy production. Although the field of sludge pretreatment has progressed significantly over the past decade, critical questions concerning the underlying microbial interactions remain unanswered. In this study, a metagenomic approach was adopted to investigate the microbial composition and gene content contributing to enhanced biogas production from sludge subjected to a novel pretreatment method (maintaining pH at 10 for 8 days) compared to other documented methods (ultrasonic, thermal and thermal-alkaline).

**Results:**

Our results showed that pretreated sludge attained a maximum methane yield approximately 4-fold higher than that of the blank un-pretreated sludge set-up at day 17. Both the microbial and metabolic consortium shifted extensively towards enhanced biodegradation subsequent to pretreatment, providing insight for the enhanced methane yield. The prevalence of *Methanosaeta thermophila* and *Methanothermobacter thermautotrophicus*, together with the functional affiliation of enzymes-encoding genes suggested an acetoclastic and hydrogenotrophic methanogenesis pathway. Additionally, an alternative enzymology in *Methanosaeta* was observed.

**Conclusions:**

This study is the first to provide a microbiological understanding of improved biogas production subsequent to a novel waste sludge pretreatment method. The knowledge garnered will assist the design of more efficient pretreatment methods for biogas production in the future.

## Background

Activated sludge technology is currently the most broadly-implemented biological method for biomass conversion in wastewater treatment plants (WWTPs) [1]. However, vast quantities of highly organic waste activated sludge (WAS) is produced during this process, and this by-product mass continues to increase with the expansion of population and industry [1-4]. Sludge disposal by landfill or incineration may no longer be feasible in the near future due to land scarcity, high waste charge and increasingly stringent environmental control regulations [4,5]. As a result, the strategy for sludge management is shifting towards its re-utilization as a potential source for renewable energy [6-8]. In this regard, the anaerobic digestion process represents an attractive means of sludge reduction while producing renewable energy in the form of biogas [8,9]. Identifying efficient ways to improve methane production, a major biogas product from anaerobic digestion, has now become a topic of interest for numerous researchers [3,10,11].

The performance of an anaerobic digestion system has been shown to be tied closely to its microbial community structure [12]. Methane production from WAS is a complex, multi-step process which involves multiple syntrophic interactions within the microbial consortium [13]. Complex compounds (polysaccharides, proteins, nucleic acids, and lipids) are first converted to oligomers and monomers through the action of extracellular hydrolytic enzymes produced by the primary fermenting bacteria [14]. Subsequently, the intermediate products are further transformed into acetate, carbon dioxide, hydrogen and formate by secondary fermenters [14]. The final methanogenesis step is then conducted by methanogenic archaea, whose energy substrates are highly restricted to acetate, H_2_, CO_2_, formate or certain C_1_ compounds [15]. To improve methane yield from sludge, enormous research efforts have been devoted to the development of pretreatment methods to accelerate sludge hydrolysis, including thermal [16], thermal-alkaline [17], ultrasonic [18], mechanical and thermo-chemical methods [19]. Although the field of pretreatment research has progressed significantly in the past decade, many significant questions related to their effects on the underlying microbial interactions remain unanswered.

As the field of pretreatment method research is nearing a threshold, the accomplishments of the past are pushing on the door of microbiology to provide new insights [20,21]. In this study, we aimed to investigate the microbial composition and gene content of sludge subjected to our novel pretreatment method (maintaining pH at 10 for 8 days) which leads to significantly enhanced methane generation compared to other documented methods (ultrasonic, thermal and thermal-alkaline) [4]. It was reported in our previous study that both sludge hydrolysis and short-chain fatty acids (SCFAs; eg. acetic, butyric and propionic acid) accumulation were significantly enhanced when WAS was anaerobically fermented under the condition of pH 10 for 8 days [22]. This phenomenon was suspected to be due to biotic factors rather than abiotic ones (e.g. alkaline hydrolysis) since much higher SCFAs accumulation and enzyme activities were observed in un-autoclaved sludge compared to autoclaved sludge [22]. Further investigation by our group suggested that the solubilization of the sludge matrix, usually a hydrolysis event by the embedded extracellular enzymes, may contribute to the significant SCFAs improvement [23].

In this study, a shotgun metagenomic approach was chosen to study potential shifts in microbial communities and/or gene contents that could help explain elevated productions of methane under our novel pretreatment method [24,25]. The latest advances in pyrosequencing technology afford new opportunities to undertake such metagenomic studies to explore the dynamics of microbial communities in time, space or under fluctuating environmental conditions with un-precedented levels of microbial diversity coverage and depth [26-28]. In addition to elucidating the microbiology underpinning the sludge pretreatment process [29], our study sought to improve knowledge of the diversity and physiology of participating syntrophs and methanogens, as well as the mechanism behind the establishment and maintenance of mutualistic cooperation. This knowledge will help to establish a better control over the hydrolysis and methanogenic processes, and promote pretreatment as part of a pertinent strategy for sludge management in WWTP. To the best of our knowledge, this study is the first to adopt a whole genome shotgun approach to link the knowledge between microbiology and engineering of sludge pretreatment methods. Additional statistical soundness was conferred to the surmised conclusion, as the comparative analysis was conducted between meta-datasets generated by the same sequencing method, from well-characterized experimental designs which only differ in known parameters by manipulation.

## Results and discussion

### Enhanced methane production by using a newly devised pretreatment strategy

**Figure 1:**
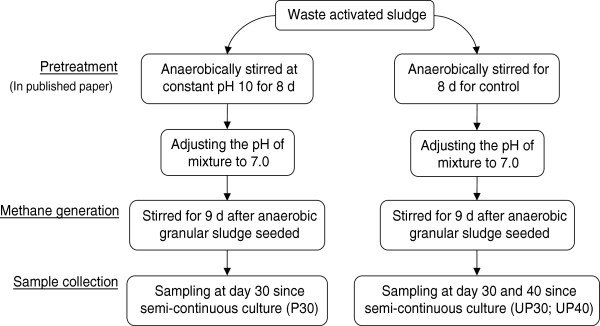
Sample preparation (P30, UP30 and UP 40) from pretreated and un-pretreated sludge bioreactors.

**Figure 2:**
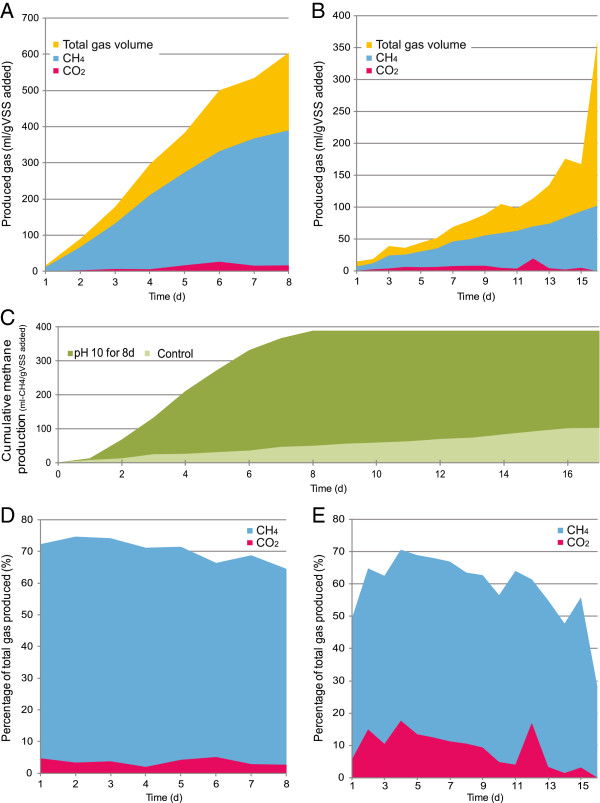
**Biogas production profile of pretreated and un-pretreated sludge bioreactors.** Efficiency of biogas production in (**A**) the pretreated sludge bioreactor is significantly higher than (**B**) the un-pretreated sludge bioreactor, giving rise to a 7.71-fold difference in the produced volume at day 8. (**C**) The cumulative methane production was 389.8 mL-CH_4_/g VSS-added using pretreated sludge as substrate, 3.78 times of that with un-pretreated sludge (103.2 mL-CH_4_/g VSS-added) as substrate. Figure 2 (**D-E**) display methane and carbon dioxide percentages produced by (**D**) pretreated and (**E**) un-pretreated sludge bioreactor, respectively; while methane is the major biogas component in both reactors, the methane content was increased by 19.3% with a decrease in carbon dioxide content by 60.9% after pretreatment.

Collected WAS was subjected to a novel pretreatment method (maintaining pH 10 for 8 days) [4], and its effects on methane production enhancement was characterized in this study. Figure 1 illustrates the experimental flowchart of obtaining end samples from the biogas-producing anaerobic digesters. Pretreated sludge for 30 days (P30), un-pretreated sludge for 30 days (UP30), or 40 days (UP40) were used as substrate. Pretreated sludge was significantly more effective in producing biogas, as total gas volume produced on day 8 (604.32 ml/g VSS-added) is 7.71 and 1.67 times of that in the un-pretreated sludge bioreactor on day 8 (78.43 ml/g VSS-added) and day 16 (360.99 ml/g VSS-added), respectively (Figure 2A-B). Methane production was 389.8 mL-CH4/g VSS-added using pretreated sludge as a substrate, which was 3.78 times that of un-pretreated sludge (103.2 mL-CH_4_/g VSS-added) (see Figure 2C). The generated methane on average constituted 70.5% and 59.1% of the total generated gas composition in the two respective bioreactors, while the rest was primarily CO_2_ (pretreated sludge, 3.6%; un-pretreated sludge, 9.2%) and other small amounts of N_2_, H_2_, NH_3_ and H_2_S (data not shown) (Figure 2D-E). It is worthwhile to note that the methane content was increased by 19.3%, and carbon dioxide content was decreased by 60.9% after the pretreatment. The reproducibility of this new pretreatment strategy validated the experimental success seen before and its technical robustness. This warranted investigation of biological mechanisms behind this significant bioreactor improvement in performance.

### Biogas producing microbial community residing in anaerobic digester inferred by metagenome sequencing

To analyze biogas-producing microbes residing in our studied bioreactors in terms of community structure, gene content, metabolic capabilities and the role of specific organisms in biogas formation, a metagenomic approach using the 454 pyrosequencing was used. Statistical data summarizing the output sequencing quantity of the three independent runs of P30, UP30 and UP40 is given in Table 1. To detect differentially abundant features between the microbial communities, we used the Meta Genome Rapid Annotation using Subsystem Technology (MG-RAST) analysis pipeline, which has shown to be applicable to various metagenomic data performing taxonomic classification as well as functional annotation [30].

**Table 1:** Statistics of 454 GS Junior pyrosequence datasets presented in this study

**Sample**	**P30**	**UP30**	**UP40**
Pretreatment	pH10 for 8d	Un-pretreated	Un-pretreated
Semi-continuous culture	30 d	30 d	40 d
Sequences count	151,676	114,694	134,522
Mean sequence length	392±120 bp	289±99 bp	425±115 bp
Archaea	8.32%	12.13%	15.13%
Bacteria	85.46%	81.82%	79.10%
Eukaryota	0.45%	0.30%	0.48%
Viruses	0.01%	0.02%	0.02%
other sequences	0.01%	0.01%	0.00%
unassigned	5.75%	5.72%	5.28%

Legends: By taxonomic classification based on M5NR database, Bacteria (82.13% on average) and Archaea (11.86% on average) are the dominant domains, with variable proportions in the bioreactor samples. UP 30 and UP40 are collected from same reactor at different time since semi-continuous culture.

Composition of the biogas-producing microbial community was obtained by taxonomic classification of reads base on the M5NR database on the MG-RAST platform. In all three reactor samples, Bacteria were the dominant superkingdom (Table 1). Domain-based allocation was primarily assigned to Bacteria (82.13% on average) and Archaea (11.86% on average), whilst the assigned reads to Eukaryota and Viruses altogether accounted for less than half a percent (Table 1). The bacterial proportion of the microbial community in P30 was the highest (85.46%) followed by UP30 (81.82%) and UP40 (79.10%). As for the Archaea domain, it ranged from 8.32% in P30, to 12.13% in UP30, and to 15.13% in UP40. The clustering pattern shown in Additional file 1 provides a graphical representation of the overall taxonomic similarity—the microbiomes of the unpretreated sludge bioreactors (UP30 and UP40) displayed high resemblance, in which the mature unpretreated sludge bioreactor (UP40, operated for longer time) is comparatively closer to the significantly diverse pretreated sludge bioreactor. The result provided the first insight into both the temporal dynamic and the enormous impact of pretreatment on microbiology. Downstream analyses focused on the Bacteria and Archaea domains (unless otherwise specified, the percentages below are representative of the identified reads within each domain per independent run).

**Figure 3:**
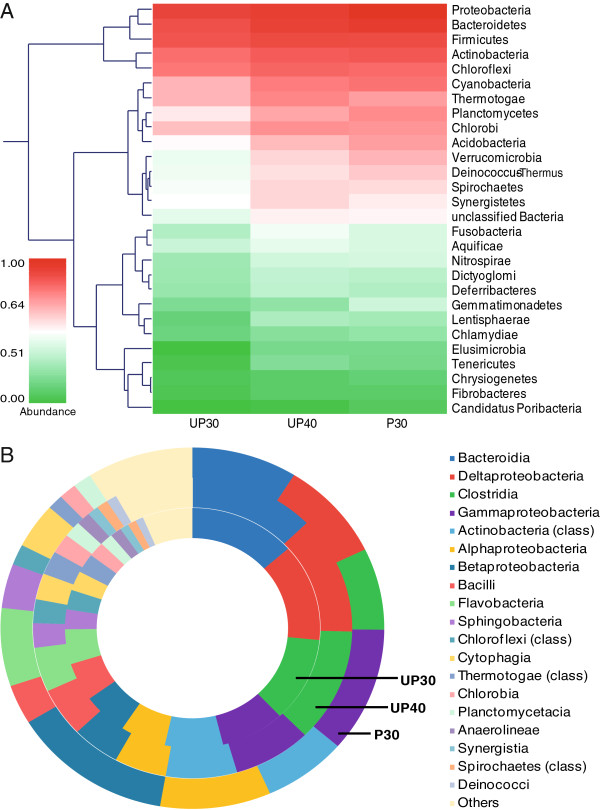
**Bacterial community in pretreated and un-pretreated sludge bioreactor at day 30 and 40.** (**A**) Proteobacteria, Bacteroidetes, Firmicutes, Actinobacteria and Chloroflexi represent top abundant phyla, all account for over 0.7 of the bacterial reads (normalized between 0 and 1) in the bioreactors. Abundance is displayed by both colour scheme and clustering dendrogram. (**B**) Bacterial consortiums which represent an anaerobic digestion community were manifested with overlapped dominant lineages in the bioreactors, with over-represented α-, β- and γ-proteobacteria in the sludge bioreactor consequent to the pretreatment (compare inner and outermost rings).

Within the Bacteria domain, the top abundant phyla were Proteobacteria (UP30, 32.17%; UP40, 30.45%; P30, 42.28%), Bacteroidetes (UP30, 22.91%; UP40, 22.25%; P30, 23.38%), Firmicutes (UP30, 16.54%; UP40, 17.65%; P30, 10.97%), Actinobacteria (UP30, 7.13%; UP40, 6.94%; P30, 6.83%) and Chloroflexi (UP30, 5.00%; UP40, 5.21%; P30, 3.37%), they collectively account for over 0.7 of the bacterial reads (normalized between 0 and 1) for each of the three bioreactors (Figure 3A). Proteobacteria, Bacteroidetes and Firmicutes have been reported to be dominant phyla as well in similar analysis of anaerobic digestion of sludge [9]. Further resolution at the class level revealed that the microbial compositions overlapped between the bioreactor samples. To be more precise, the dominant bacterial lineages presented in this study were related to anaerobic digestion, including Bacteroidia (UP30, 13.50%; UP40, 12.69%; P30, 8.90%), δ-proteobacteria (UP30, 13.08%; UP40, 12.71%; P30, 8.93%), Clostridia (UP30, 11.42%; UP40, 12.21%; P30, 7.31%), γ-proteobacteria (UP30, 8.12%; UP40, 7.90%; P30, 11.18%), Actinobacteria (class) (UP30, 7.37%; UP40, 7.20%; P30, 7.06%), α-proteobacteria (UP30, 5.78%; UP40, 5.28%; P30, 9.33%), β-proteobacteria (UP30, 5.73%; UP40, 4.86%; P30, 13.58%), Bacilli (UP30, 5.16%; UP40, 5.25%; P30, 3.53%), Flavobacteria (UP30, 4.70%; UP40, 4.76%; P30, 6.98%) and Chloroflexi (class) (UP30, 2.65%; UP40, 2.56%; P30, 1.84%) (Figure 3B).

With time, there was moderate temporal variation in the relative abundances of these bacterial members in the anaerobic digester fed with un-pretreated sludge (UP30 and UP40). While the relative proportion of these communities remained comparable between UP30 and UP40, a larger extent of differences could be observed when P30 was taken into comparison for certain lineages. The over-represented bacterial members in P30 included α-proteobacteria (9.33%), β-proteobacteria (13.58%) and γ-proteobacteria (11.18%); which are respectively 69.06%, 158.21% and 39.60% more than UP30 and UP40 on average. Overall, these sequences were originated from the WAS substrate, AGS inoculum and the biogas-producing symbiont within the system, hence the differences observed in their relative abundance were attributable to the sole variable of either using pretreated or un-pretreated sludge in the bioreactors. These results suggested that the bacterial communities that underlie the anaerobic digesters were dynamic, and they responded rapidly to the pretreated sludge substrate and change substantially over time.

Based on the phylogenetic affiliation of the metagenomic sequences, it is possible to form hypotheses regarding the metabolic functions of the groups [9]. Proteobacteria are microorganisms involved in the initial steps of degradation, studies have shown that they are the main consumers of propionate, butyrate and acetate [31]. Bacteroidetes are known proteolytic bacteria, responsible for the degradation of protein and subsequent fermentation of amino acids into acetate for acetoclastic methanogens [31,32]. Concerning Firmicutes, they are syntrophic bacteria which degrade volatile fatty acids such as butyrate [31]. The H_2_ generated from this process could be then uptaken by the hydrogenotrophic methanogens [33]. The bacterial class Clostridia is frequently found in co-culture with other species in biomasss conversion systems and it includes a number of anaerobic species that are commonly associated with the decomposition of lignocelluloses and municipal solid waste [34,35]. As for Chloroflexi, this group is often found in various wasterwater treatments such as anaerobic digesters and biological nutrient removal processes; their potential role in carbohydrate degradation has been reported in several studies [31,36-38]. Overall, the resident bacteria manifested in the bioreactors represented a biomass decomposing community.

Archaeal representatives were less diverse and consisted of only three major families of methanogens, belonging to Methanosarcinaceae, Methanobacteriaceae and Methanosaetaceae (Table 2). The predominance of selected methanogen lineages in the Archaeal domain (represented as percentage of total identified reads in Archaea) has been observed in both production and laboratory-scale biogas reactors [23,39,40], and concluded in a recent meta-analysis of the collective microbial diversity [41,42]. At the genus level, *Methanosarcina* was determined to be the most abundant methanogen in all three bioreactors (13.51% on average—UP30, 15.10%; UP40, 13.32%; P30, 12.10%), while *Methanosaeta* (10.67% on average—UP30, 13.13%; UP40, 10.31%; P30, 8.56%) and *Methanothermobacter* (10.35% on average— UP30, 10.73%; UP40, 9.41%; P30, 10.91%) contributed to slightly lesser portions. Anaerobic digesters are typical habitats to these three genera of methanogens [43], and the enrichment of *Methanosarcina* species was in congruence with other evaluation studies on primary sludge and WAS anaerobic digesters [44,45]. The observation of a lesser portion of *Methanothermobacter* was expected, as the operating condition of the bioreactor (approximate 35°C) was not in favour of this thermophilic Archeon’s proliferation (55-65°C) [43].

**Table 2:** Relative abundance of Archaea in pretreated and un-pretreated sludge bioreactors at day 30 and 40

**Archaeal taxonomic affiliation**	**Percentage of all Archaeal reads**		
**(phylum/ family/ genus/ species)**	**UP30**	**UP40**	**P30**
Euryarchaeota	97.60%	95.60%	94.86%
Methanosarcinaceae	23.11%	20.56%	19.14%
*Methanosarcina*	15.10%	13.32%	12.10%
Methanobacteriaceae	20.81%	18.61%	21.44%
*Methanothermobacter*	10.73%	9.41%	10.91%
*Methanothermobacter thermautotrophicus*	9.50%	8.59%	9.94%
Methanosaetaceae	14.00%	11.03%	9.19%
*Methanosaeta*	13.13%	10.31%	8.56%
*Methanosaeta thermophila*	14.48%	12.36%	10.25%
Crenarchaeota	2.33%	3.61%	4.40%
Korarchaeota	0.00%	0.45%	0.37%
Thaumarchaeota	0.05%	0.27%	0.37%
Nanoarchaeota	0.02%	0.07%	0.00%

Legends: Archaeal domain consists three major families belonging to Methanosarcinaceae, Methanobacteriaceae and Methanosaetacea, while *Methanosarcina*, *Methanosaeta* and *Methanothermobacter* represent top abundant methanogens on the genus level. The decrease in abundance of *Methanosarcina*-related species in P30 was correlated with an enhanced bioproduction of acetate in the pretreated sludge. Except for phylum level, only candidates of abundance >8% are presented. Species are represented as percentage of all methanogens.

*Methanosaeta* species were observed to be more abundant in samples of bioreactor digesting un-pretreated sludge (UP30; 13.13%) than that with pretreated sludge (P30; 8.56%), but the population decreases with time (UP40; 8.56%). A similar observation was found in an earlier surveillance of the methanogenic population dynamics in anaerobic digesters, where the hitherto abundant *Methanosaeta* population decreased rapidly as the acetate concentration increased [12]. As acetoclastic methanogens, both *Methanosarcina* and *Methanosaeta* are able to split acetate, oxidize the carboxyl-group to CO_2_ and reduce the methyl group to CH_4_[43]. At the same time, while *Methanosarcina* thrive in environments with high acetate concentration; lower acetate concentrations benefit the dominance of *Methanosaeta* owing to their high affinity for acetate [12,46-48]. As aforementioned, an enhanced bioproduction of acetic acid was reported in the pretreated sludge [4], the revealed shift in the methanogenic community could therefore be interpreted as an ecological consequence [30,49,50] of the sludge pretreatment.

### Methane production via acetoclastic and hydrogenotrophic pathways

**Figure 4:**
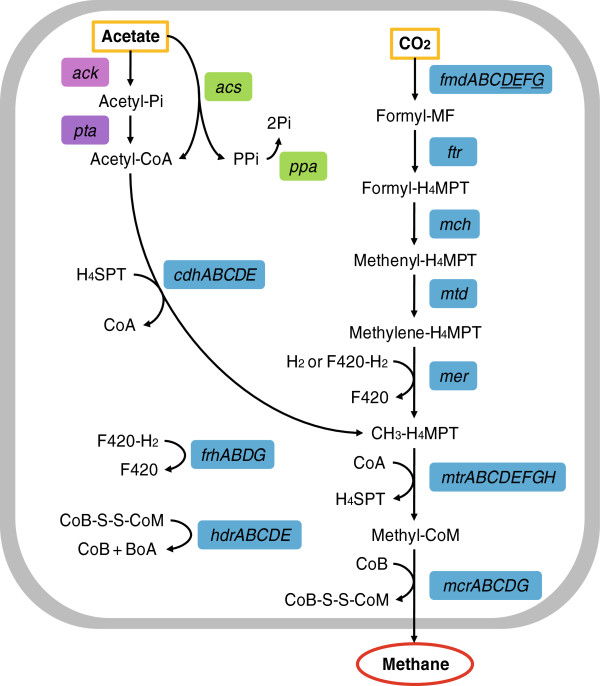
**Reconstruction of methanogenesis pathways occurring in the bioreactors using identified genes.** Positive identifications in meta-datasets are shown in colored boxes, with negative identifications shown underlined. Gene candidates for the formation of acetyl-CoA from acetate in the *Methanosaeta* bin are displayed in green boxes, and negative identifications particular to this bin are shown in purple boxes. See Additional file 2 for full form of abbreviated names and detailed identification counts.

To identify the methane-producing organisms in the bioreactor samples, taxonomic and functional affiliation of the metagenomic reads were evaluated in parallel (represented as percentage of total identified methanogens). On the species level, it is common to all three bioreactor samples that *Methanosaeta thermophila* (12.36% on average—UP30, 14.48%; UP40, 12.36%; P30, 10.25%) and *Methanothermobacter thermautotrophicus* (9.34% on average—UP30, 9.50%; UP40, 8.59%; P30, 9.94%) are the over-represented methanogens (Table 2). Here, *Methanosarcina* species were not represented as one of the top dominant methanogens (13.51% on average—UP30, 15.10%; UP40, 13.32%; P30, 12.10%), this observation was linked to the intra-divergence of each family [9]. *M. thermophila* is an obligate methanogen which consumes acetate only [51], whereas *M. thermautotrophicus* conserves energy by using H_2_ to reduce CO_2_ to CH_4_[33,52]. While the pH was adjusted to be within the optimum range (7.0) for the two dominate methanogens after pretreatment (Figure 1), it was interesting to detect the prevalence of the thermophilic *M. thermautotrophicus* in the bioreactors that operated at mesophilic temperature [53]. At the same time, functional enzyme-encoding genes for the two methanogenesis pathways were identified with reference to KEGG and Metacyc pathway database entries (Figure 4, Additional file 2) [54,55]. Nonetheless, based on these results, it was evinced that the production of methane in the studied bioreactors was performed by methanogenic Archaea via acetoclastic and hydrogenotrophic pathways.

Acetoclastic and hydrogenotrohpic pathways are indeed common methanogenesis pathways reported in various reports [42,56]; the affirmation of these pathways permitted subsequent in-depth investigation of the encompassed enzymology. The first step in acetoclastic methanogenesis is the formation of acetyl-CoA from acetate [51]. Analysis of the recently completed genome of *Methanosaeta thermophilia* confirmed that the majority of the acetoclastic pathway are similar for *Methanosaeta* and *Methanosarcina*, except the enzymes employed for the first step of catalyzation [51]. It was proposed that acetoclastic methanogenesis in *Methanosaeta* proceeded with a modified version of the pathway compared with *Methanosarcina*, which utilizes the acetate kinase/phosphotransacetylase pathway to convert acetate to acetyl-CoA [57,58]. Taking *M. thermophila* as an example, its genome does not include a readily identifiable acetate kinase, and it has been postulated that this archeon utilizes an acetate transporter coupled with acetyl-CoA synthetases to convert acetate to acetyl-CoA, and the hydrolysis of pyrophosphate by inorganic pyrophosphatase (PPase) drives this reaction forward [51]. In this study, the analysis of metagenomic datasets using SEED annotation indicated the presence of acetyl-CoA synthetases (EC 6.2.1.1) and inorganic pyrophosphatase (EC 3.6.1.1) in the compiled *Methanosaeta* bin. While the sequencing depth might account for the absence of genes for the acetate kinase/phosphotransacetylase pathway, the discovery of these two enzymes advocated an alternative pathway in acetotrohpic methanogenesis for *Methanosaeta*[41]. Results from a recent study on a terephthalate-degrading bioreactor supported this hypothesis [41].

### Functional affiliation related to higher methane production after sludge pretreatment

**Figure 5:**
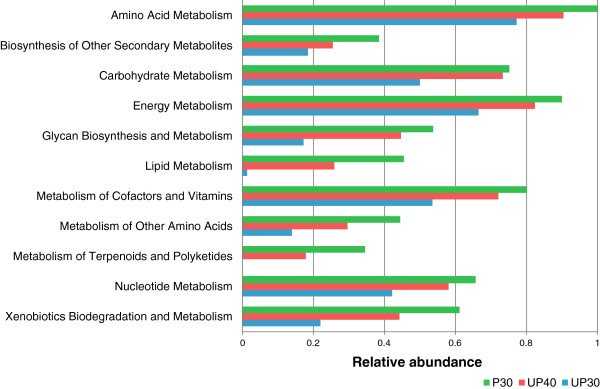
**Relative abundance of functional reads affiliated to metabolisms associated to higher methane production using KO database.** Expression of these faculties in the un-pretreated sludge bioreactor gradually elevated over time (compare UP30 and UP40), and were highest in pretreated-sludge bioreactor (P30).

Degradation of the highly organic polymers represents the first and overall rate-limiting step for the mineralization of organic matter in activated sludge and anaerobic digested sludge treatment systems [59-61]. In our work, the studied pretreatment involved alteration of pH, which is an important parameter affecting both bacterial activity and metabolite pathways. KO-based annotations were used to understand how these phylogenetic trends could be used to predict the metabolic potential of these microbes. Figure 5 shows the subsystems that are related to higher methane production, including metabolism of amino acids, energy, carbohydrates, nucleotides, lipids, cofactors and vitamins, xenobiotics, as well as the fermentation of different substrates. These results revealed a general elevated expression of these faculties in the bioreactors fed with un-pretreated sludge over time (compare UP30 and UP40), whereas these levels were highest in the bioreactor digesting pretreated sludge (P30). Distribution of the functional systems was most divergent in P30, which showed predominance in metabolism consistent with a community shifted towards an enhanced biomass degradation metabolism. Herewith, the downstream analysis focused on the degradation of carbohydrates for a number of reasons. Firstly, the matrix of extra-cellular polymeric substances (EPS) that crosslink cells together remains the primary solid part of biological sludge, of which the polysaccharide is the predominant component [62-64]. Secondly, complex carbohydrate is the commonplace recalcitrant in the hydrolysis process, and bioprospecting for carbohydrate active enzymes is a topic of interest in metagenomics [65]. As a result, the focus of our study was hence placed on the degradation of this dominant recalcitrant component. (Please see Additional file 3 for further insights towards protein and lipid degradation.)

As sequencing technology provides access to a remarkable array of microbial functional capacity, sequence-based data mining is an important prospect in metagenomic projects [66,67]. To understand how the microbial community mediates the solibulization of the sludge matrix, a carbohydrate-active enzyme (CAZy) characterization of the metagenomic datasets was performed after *ab initio* gene prediction [68,69]. Conventional sequence homology-based enzyme discovery introduces a bias towards the identification of candidates similar to known enzymes, rather than enzymes with low sequence identity and potentially divergent biochemical properties [66]. The entries in the CAZy database contains both experimentally verified and putative carbohydrate-active enzyme domains, hence this search strategy would be able to provide a better insight into the catalysis of biochemical reactions [68,70].

In this study, a total of 1917 and 107 gene modules were recognized across 52 glycosyl hydrolases (GH) and 9 carbohydrate binding modules (CBM) respectively (Tables 3 and 4). GH is defined as a widespread group of enzymes which hydrolyzes the glycosidic bond between carbohydrates or between that and a non-carbohydrate moiety [68]. On the other hand, CBM are contiguous amino acids within a carbohydrate-active enzyme with a discreet fold which bears the carbohydrate-binding activity [68]. In contrast to the small number of enzymes devoted to the hydrolysis of the main chain of cellulose, hemicelluloses and pectins (GH 5, 6, 7, 8, 9, 12, 44, 45, 48 and 74), all three metagenomes displayed a variety of enzymes that digest the side chains of these polymers and oligosaccharides. Families of GH 2 and 3, which contain a large range of glycosidases cleaving non-reducing carbohydrates in oligosaccharides and the side chains of hemicelluloses and pectins, were particularly abundant in the bioreactor samples (29.10% of all GH—UP30, 27.56%; UP40, 29.55%; P30, 24.20%). CBM 48 was the most dominant in the three metagenomes, it binds glycogen and is commonly found in a range of enzymes that act on branched substrates, such as the hydrolysis of glycogen, amylopectin and dextrin by isoamylase, pulluanase and branching enzyme. Overall, the number of GH hit returns appeared to increase with time in the bioreactor fed with un-pretreated sludge (468 counts in UP30; 792 counts in UP40); while the bioreactor digesting the pretreated sludge harboured 1.40 times the GH count in the control counterpart at the same time. As for CBM, P30 (51 counts) had a higher abundance than UP40 (38 counts), and made up to approximately 2.83 times that of UP30. Generally, there appeared to be a dynamic process in enzymology within the anaerobic digesters with time and substrate nature The heightened GH and CBM abundance in the bioreactor with pretreated sludge may possibly correlate with the higher sludge solubilization proposed in previous studies [23].

**Table 3:** Identification of glycoside hydrolase (GH) family classified by CAZy database

		**Number of counts**					**Number of counts**		
**CAZy GH family**	**Pfam model**	**UP30**	**UP40**	**P30**	**CAZy GH family**	**Pfam model**	**UP30**	**UP40**	**P30**
GH 1	PF00232.12	11	14	15	GH 38C	PF07748.7	20	42	15
GH 2	PF00703.15	7	27	28	GH 39	PF01229.11	2	1	6
GH 2_C	PF02836.11	18	38	31	GH 42	PF02449.9	8	18	12
GH 2_N	PF02837.12	26	61	44	GH 42C	PF08533.4	0	3	3
GH 3	PF00933.15	49	69	59	GH 42 M	PF08532.4	7	5	4
GH 3_C	PF01915.16	29	39	33	GH 43	PF04616.8	12	27	33
GH 4	PF02056.10	9	20	10	GH 44	PF12891.1	1	1	2
GH 8	PF01270.1	0	0	1	GH 4C	PF11975.1	8	18	8
GH 9	PF00759.1	9	5	7	GH 53	PF07745.7	1	1	10
GH 10	PF00331.14	4	10	11	GH 57	PF03065.1	22	38	12
GH 15	PF00723.1	2	2	7	GH 62	PF03664.7	0	1	0
GH 16	PF00722.15	11	9	9	GH 63	PF03200.10	1	1	0
GH 17	PF00332.12	0	0	1	GH 65C	PF03633.9	2	2	3
GH 18	PF00704.22	7	11	7	GH 65 m	PF03632.9	19	5	12
GH 19	PF00182.13	0	0	1	GH 65 N	PF03636.9	3	8	5
GH 20	PF00728.16	25	40	22	GH 67C	PF07477.6	2	5	1
GH 20b	PF02838.9	12	6	7	GH 67 M	PF07488.6	3	6	7
GH 25	PF01183.14	7	8	8	GH 67 N	PF03648.8	4	9	5
GH 26	PF02156.9	5	10	6	GH 76	PF03663.8	0	1	1
GH 28	PF00295.11	2	16	11	GH 77	PF02446.11	7	26	17
GH 30	PF02055.10	3	8	11	GH 81	PF03639.7	0	0	1
GH 31	PF01055.20	20	29	30	GH 88	PF07470.1	9	10	18
GH 32C	PF08244.6	0	5	0	GH 92	PF07971.6	34	49	46
GH 32 N	PF00251.14	4	4	3	GH 97	PF10566.3	15	49	26
GH 35	PF01301.13	12	4	18	GH 101	PF12905.1	7	3	7
GH 38	PF01074.16	8	26	21	GH cc	PF11790.2	1	2	2
					**Total number of counts**		**468**	**792**	**657**

Legend: Dynamic process in enzymology with time and substrate nature was observed in the bioreactors, the heightened GH and abundance (in bold) in the pretreated-sludge bioreactor were correlated with a higher sludge solubilization, and in turns a higher SCFA production.

**Table 4:** Identification of carbohydrate binding module (CBM) family classified by CAZy database

		**Number of counts**		
**CAZy CBM family**	**Pfam model**	**UP30**	**UP40**	**P30**
CBM_2	PF00553.13	0	0	1
CBM_4_9	PF02018.11	1	5	6
CBM_5_12	PF02839.8	0	2	1
CBM_6	PF03422.9	2	6	8
CBM_11	PF03425.7	0	3	1
CBM_20	PF00686.13	0	4	3
CBM_25	PF03423.7	0	1	0
CBM_48	PF02922.12	11	10	20
CBM_X	PF06204.5	4	7	11
**Total number of counts**		**18**	**38**	**51**

Legend: Dynamic process in enzymology with time and substrate nature was observed in the bioreactors, the heightened CBM abundance (in bold) in the pretreated-sludge bioreactor were correlated with a higher sludge solubilization, and in turns a higher SCFA production.

## Conclusions

The treatment and disposal of excess sludge accumulation represents a bottleneck in wastewater treatment plants worldwide due to environmental, economic, social and legal factors. Reduction of excess sludge is becoming one of the biggest challenges in biological wastewater treatment [71,72]. The anaerobic digestion process is a promising technique, along with which waste is eliminated and methane is produced as a valuable renewable energy source. Understanding the impact of pretreatment on the microbiology at different stages of anaerobic digestion is important; it ultimately impacts the performance of a bioreactor. This study represents the first attempt to gain an in-depth metagenomic perspective, with regard to taxonomic and functional aspects, on increased biogas (methane) production when a newly devised pretreatment method is used. Dual taxonomic and functional analysis indicated the microbial and metabolic consortium shifted extensively towards enhanced biodegradation, also seen in the methanogenesis pathways. Altogether, these results presented here help further microbiological understanding in sludge pretreatment research for anaerobic digestion. As microbiology is the ultimate driver for the anaerobic digestion process, this new perspective encourages a closer engagement between the engineering and microbiology knowledge pools. Further studies involving deeper sequencing of the metagenomes to characterize the decomposing and methanogenic microbiome at higher frequency and replicate number parallel to the physical-chemical characterization are required for each WAS pretreatment intervention.

## Methods

### Collection of waste activated sludge and anaerobic granular sludge

The waste activated sludge (WAS) used as substrate for methane production was collected from the secondary sedimentation tank of a municipal wastewater treatment plant in Shanghai, China. The anaerobic granular sludge (AGS) used as the inoculum for methane production was obtained from the upward-flow anaerobic sludge blanket (USAB) reactor of a food wastewater treatment plant in Yixing, China. The main features of WAS and AGS can be found in Additional file 4. The AGS was cultured in a laboratory up-flow anaerobic sludge bed (UASB) by synthetic wastewater (Additional file 5) prior to its use as the inoculum for methane production, where the use of synthetic wastewater for AGS culturing is a common practice in anaerobic digestion studies investigating the microbiology and biodegradability [4,73,74]. The hydrolytic retention time of UASB was 6 h, and the AGS concentration in UASB was approximately 29165 mg/L.

### Pretreatment of waste activated sludge

Biogas-producing, anaerobic digester samples were obtained from a reproduction of the experiment described in our previous work [4], which introduced a new sludge pretreatment strategy (pH 10 for 8 d) for enhanced methane production from WAS. During the pretreatment, the pH of the collected WAS was adjusted to 10.0 by addition of 5 M NaOH or 4 M HCl with an automatic titrator, in a tightly-sealed reactor which was mechanically stirred (80 rpm) at 35 ± 1°C for 8 d. A blank test was also conducted, in which the sludge was not pretreated but mechanically stirred for 8 d. Then the pH in all reactors was adjusted to pH 7.0, and 30 mL of AGS was added to each reactor for methane production. All reactors were sealed with rubber stoppers and mechanically stirred at 80 rpm without further pH control. The retention time of all reactors was 17 d and the organic loading rate was 1.88 kg/COD/m^3^/d. The gas generation was detected by water displacement and the methane concentration was measured by a gas chromatograph (GC-14B, Shimadzu, Japan). The analyses of VSS were conducted in accordance with APHA Standard Methods [75]. In this study, the methane yield was reported as the amount of methane generated per gram of volatile suspended solid added (mL-CH_4_/gVSS-added) unless otherwise stated. The full unit of methane production is ml-CH_4_/gVSS-added, in which the VSS-added refers to the initial VSS to facilitate comparison of methane production. Moreover, there was no SCFA in the original sludge before anaerobic fermentation, meaning that the initial VSS did not contain volatile fatty acids—hence the risk of overestimation of specific methane yield is improbable [4].

### Preparation of anaerobic digester samples

The granular samples cultured with pretreated (pH 10 for 8 d) and un-pretreated WAS were collected when the methane production rate reached the maximum on day 17, and then further cultured by a semi-continuous method to maintain the methane production rate. In replicates of three, the semi-continuous flow reactors with working volume of 1.0 L received 470 mL of pH 10 pretreated sludge and un-pretreated sludge, respectively. Everyday, 52 mL sludge mixture was manually wasted from each reactor, and 52 mL pretreated and un-pretreated sludge was added to the respective reactors; AGS was fed back to reactors by sifting the wasted sludge through a sifter with aperture of 0.2 mm. Further procedure details were described in [4].

As a stable methane generation continued to be maintained by the semi-continuous culturing method, 50 mL anaerobic digester samples were collected from the pretreatment set-ups (as pooled sample) at day 30 (P30), control set-up at day 30 (UP30) and control set-up at day 40 (UP40) for microbial studies. The two samples collected at day 30 (P30, UP30) served to characterize taxonomic and functional difference between pretreated and unpretreated samples. Both sludge bioreactor were seeded with the same synthetic wastewater cultured AGS substrate, and later gradually replaced by the experimental sludge (pretreated or unpretreated WAS) completely as the batch experiment continued. Since both bioreactors received the same inoculums, therefore the only existing variable was the pretreatment, ascribing to the observed difference between the microbiomes. As for UP40, this additional un-pretreated sample at day 40 was prepared to provide further insight of the microbial dynamic in response to time. Figure 1 shows the experimental scheme for the preparation of the anaerobic digester samples.

### DNA extraction and 454 sequencing

Samples (P30, UP30 and UP40) were kept at −20°C and thawed once only for nucleic acid extraction using PowerSoil® DNA Isolation Kit (Mobio, Carlsbad, USA), conducted within 24 hours of sample collection. DNA quantification was performed using Nanodrop 2000 spectrophotometer (Nanodrop Technologies, Wilmington, USA). Sequencing of total DNA derived from each was done by the shotgun sequencing approach on the 454 GS Junior system, and the sequencing quality was maintained by 454 propriety software. The raw sequence reads are submitted under the Sequence Read Archive (SRA) accession number SAMN01924662(P30), SAMN01924663 (UP30), SAMN01924664 (UP40).

### Metagenome analysis

To obtain a quantitative picture of the taxonomic composition, sequence datasets were characterized without a prior assembly step [56]. The unassembled sequence reads were submitted to the online metagenome analysis tool Metagenome Rapid Annotation using Subsystem Technology (MG-RAST analysis pipeline 3.0) [76]. Following the quality trimming with default setting, taxonomic and functional profiling was performed using the M5 non-redundant protein database (M5NR) and KEGG Orthology (KO) reference database [54] Parameters used were e-value cutoff of 1 × 10^-5^, minimum alignment length of 50 base pairs, and a minimum percentage identity at 50%. Relative abundance of the Archaeal and Bacterial groups were determined by the percentages of respective reads over total assigned reads. Parallel processing was performed similarly on Megan4 to confirm the clustering pattern of taxonomic structure [77]. Only the taxonomic and functional annotation provided by MG-RAST was relied upon for all subsequent analysis.

To identify important carbohydrate- metabolism related gene candidates, pyrosequence reads were first subjected to gene prediction by a heuristic approach using MetaGeneMark 1.0 [69]. Next, the amino acid sequences were screened against catalytic domains (CD) characteristic for particular glycoside hydrolase (GH) families or for a particular carbohydrate binding module (CBM) as classified by the CAZy database [68]. HMM models were downloaded from Pfam database version 25.0 and used as a database for pfam_scan [78] (e-value cutoff of 1x10^-4^). Methanogenesis related gene candidates were identified with reference to KEGG and Metacyc pathway database [54,55].

## Abbreviations

AGS: Anaerobic granular sludge; CBM: Carbohydrate binding modules; CAZy: Carbohydrate-active enzyme; CD: Catalytic domains; GH: Glycosyl hydrolases; PPase: Inorganic pyrophosphatase; SRA: Sequence Read Archive; SCFAs: Short-chain fatty acids; USAB: Upward-flow anaerobic sludge blanket; VSS: Volatile suspended solid; WAS: Waste activated sludge; WWTPs: Wastewater treatment plants

## Competing interests

The authors declare that they have no competing interests.

## Authors’ contributions

MTW performed the sample preparation, sequence analyses, data interpretation and compiled the manuscript. DZ carried out the anaerobic digestion and physical/chemical characterization. JL compiled the computational analyses tools. RKHH performed the next-generation sequencing. HMT, MSB and TJP contributed to data interpretation and manuscript’s final revision. FCL and YC conceived and coordinated the study. All authors read and approved the final manuscript.

## Authors’ information

FCL is a Full Professor at School of Biological Sciences, former Dean of Faculty of Science, Board member of Initiative on Clean Energy and Environment, The University of Hong Kong, and Fellow of American Association for the Advancement of Science (AAAS). RKHH is a post-doc, JL, HMT, MSB and TJP are PhD students, MTW is an MPhil student in the same institution. YC is a Full Professor and DZ is a post-doc at State Key Laboratory of Pollution Control and Resources Reuse, School of Environmental Science and Engineering, Tongji University.

## Supplementary Material

Additional file 1 **Taxonomic clustering of microbiome in pretreated and un-pretreated sludge bioreactor at day 30 and 40.** The clustering pattern revealed a proximate microbial resemblance between un-pretreated sludge bioreactors and the highly divergent community in pretreated sludge bioreactor impacted by the pretreatment. Click here for file

Additional file 2 **Methanogenesis gene identifications in pyrosequencing meta-datasets UP30, UP40, P30 using KO and SEED reference database.** Genes related to methanogenesis were identified by comparing the pyrosequence meta-datasets UP30, UP40 and P30 with KO and SEED database. Negative identifications were indicated as ‘-’. Click here for file

Additional file 3 **Detailed profile related to (a) amino acid and (b) fat metabolism in the meta-datasets UP30, UP40 and P30 using KO reference database.** Expression of these metabolism faculties in the un-pretreated sludge bioreactor gradually elevated over time (compare UP30 and UP40), and were highest in pretreated-sludge bioreactor (P30). Label: [KO Map number] [metabolism detail] [path number]. Click here for file

Additional file 4 **Properties of WAS and AGS after settling.** Description of data: All values are expressed in mg/L except pH. The data are the averages and their standard deviations in duplicate tests. Click here for file

Additional file 5 **Synthetic wastewater composition.** Description of data: Chemical characterization of synthetic wastewater. Click here for file
