# Supplementary figures and images for: Towards a metagenomic understanding on enhanced biomethane production from waste activated sludge after pH 10 pretreatment
Source: Biotechnol Biofuels. 2013 Mar 19;6:38. doi: 10.1186/1754-6834-6-38 (PMC3607842; doi:10.1186/1754-6834-6-38)

UP40

P30

UP30

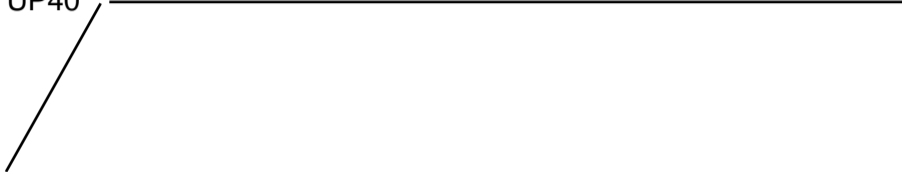

Supplement: Additional file 1 — Taxonomic clustering of microbiome in pretreated and un-pretreated sludge bioreactor at day 30 and 40. The clustering pattern revealed a proximate microbial resemblance between un-pretreated sludge bioreactors and the highly divergent community in pretreated sludge bioreactor impacted by the pretreatment. [file 1754-6834-6-38-S1.pdf]

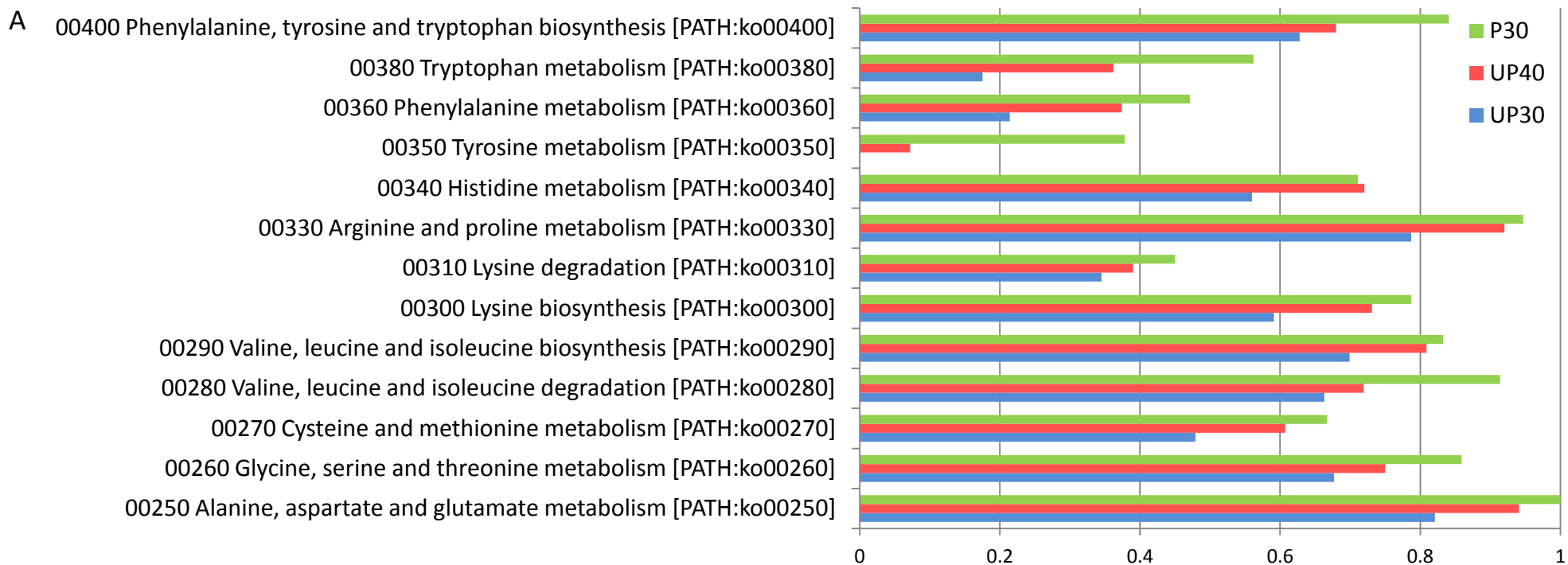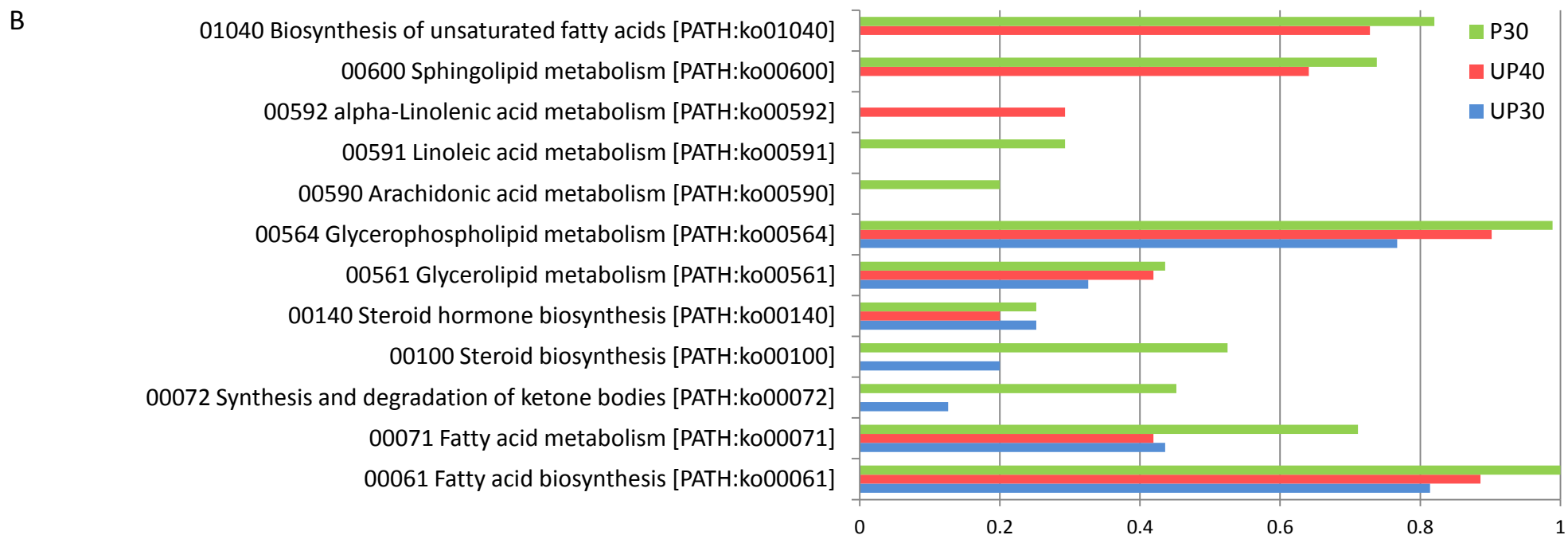

Supplement: Additional file 3 — Detailed profile related to (a) amino acid and (b) fat metabolism in the meta-datasets UP30, UP40 and P30 using KO reference database. Expression of these metabolism faculties in the un-pretreated sludge bioreactor gradually elevated over time (compare UP30 and UP40), and were highest in pretreated-sludge bioreactor (P30). Label: [KO Map number] [metabolism detail] [path number]. [file 1754-6834-6-38-S3.pdf]
